# Case report: Thrombotic microangiopathy concomitant with macrophage activation syndrome in systemic lupus erythematosus refractory to conventional treatment successfully treated with eculizumab

**DOI:** 10.3389/fmed.2022.1097528

**Published:** 2023-01-09

**Authors:** Makoto Yamaguchi, Masashi Mizuno, Fumiya Kitamura, Shiho Iwagaitsu, Hironobu Nobata, Hiroshi Kinashi, Shogo Banno, Akimasa Asai, Takuji Ishimoto, Takayuki Katsuno, Yasuhiko Ito

**Affiliations:** ^1^Department of Nephrology and Rheumatology, Aichi Medical University, Nagakute, Japan; ^2^Renal Replacement Therapy, Nagoya University Graduate School of Medicine, Nagoya, Japan; ^3^Department of Nephrology and Rheumatology, Aichi Medical University Medical Center, Okazaki, Japan

**Keywords:** thrombotic microangiopathy, macrophage activation syndrome, systemic lupus erythematosus, neuropsychiatric systemic lupus erythematosus, eculizumab

## Abstract

Thrombotic microangiopathy (TMA) is a rare but life-threatening complication of systemic lupus erythematosus (SLE). Macrophage activation syndrome (MAS) is also a rare, life-threatening hyperinflammatory condition that is comorbid with SLE. However, the association between TMA and MAS in patients with SLE has rarely been assessed, and the difficulty of diagnosing these conditions remains prevalent. The efficacy of eculizumab has been reported for SLE patients whose conditions are complicated with TMA. However, no study has investigated the therapeutic efficacy of eculizumab for TMA concomitant with SLE-associated MAS. Herein, we report the first case of TMA concomitant with SLE-associated MAS that was initially refractory to conventional immunosuppressive therapy but showed remarkable recovery after eculizumab treatment. Furthermore, we evaluated serum syndecan-1 and hyaluronan levels, which are biomarkers of endothelial damage. We found that these levels decreased after the administration of eculizumab, suggesting that TMA was the main pathology of the patient. This case illustrates that it is important to appropriately assess the possibility of TMA during the course of SLE-associated MAS and consider the use of eculizumab as necessary.

## 1. Introduction

Thrombotic microangiopathy (TMA) is a rare but life-threatening condition characterized by microangiopathic hemolytic anemia (MAHA), thrombocytopenia, and varying degrees of organ damage, including renal failure and central nervous system (CNS) dysfunction. TMA is classified as primary TMA when genetic and acquired defects in complement proteins are its primary drivers [complement-mediated TMA or atypical hemolytic uremic syndrome (aHUS)] or secondary TMA when complement activation occurs in the context of other disease processes, such as infection, autoimmune disease, malignant hypertension, malignancy, transplantation, pregnancy, and drugs (1–6). Barring rare complications, a variety of autoimmune diseases have been associated with TMA (7), and systemic lupus erythematosus (SLE) is known to be one of the most commonly acquired causes of TMA (occurrence ranges from 3 to 9% in patients with SLE).

Macrophage activation syndrome (MAS) is also a life-threatening hyperinflammatory condition that rarely complicates autoimmune diseases and belongs to the spectrum of hemophagocytic lymphohistiocytosis (HLH) (8). The pathophysiological hallmark of MAS is a dysfunctional immune response, which leads to excessive activation and expansion of T lymphocytes and macrophages, resulting in the cytokine storm syndrome (9, 10). The main clinical features of MAS are unremitting fever, hepatosplenomegaly, generalized lymphadenopathy, CNS dysfunction, and hemorrhagic manifestations. MAS is a relatively uncommon complication of SLE in adults, with an occurrence reported to range from 0.9 to 4.6% (11). Although the mortality rate of SLE patients with MAS is high (12), early diagnosis of MAS in SLE is not easy since there are no uniform diagnostic criteria, and the clinical characteristics of MAS-associated SLE, as well as active SLE, are similar, making the diagnosis of MAS more difficult (12, 13).

Meanwhile, the association between TMA and MAS has rarely been assessed. Only a few case reports have previously described the coexistence of TMA and MAS as a complication of hematopoietic stem cell transplantation (13, 14), renal transplantation (15), autoimmune disease in children (mainly systemic juvenile idiopathic arthritis) (16), and complement-mediated TMA (17). Therefore, little is also known about the relationship between TMA and MAS in SLE patients, and it is challenging to recognize TMA in a timely manner in the diagnosis of MAS because the clinical characteristics of TMA are similar to those of MAS (18, 19). Therefore, the possible coexistence of TMA and MAS in SLE may be underrecognized (16).

Eculizumab is a humanized monoclonal antibody directed against complement C5. It blocks the terminal alternative complementary pathway by binding to the C5 convertase site, preventing the formation of C5b-9 (a membrane attacking complex) and C5a (a potent anaphylatoxin) (20). The efficacy of eculizumab has been reported for SLE with lupus nephritis complicated by TMA (21), leading to the recovery of renal function. However, no study has investigated the therapeutic efficacy of eculizumab for TMA concomitant with SLE-associated MAS.

To the best of our knowledge, this is the first report of a patient with TMA concomitant with SLE-associated MAS who was refractory to conventional therapy but showed a remarkable recovery after eculizumab therapy. Furthermore, we evaluated serum levels of syndecan-1 and hyaluronan, which are biomarkers of endothelial damage, to diagnose TMA and confirm that these levels decreased after the administration of eculizumab.

## 2. Case report

A 58-year-old Japanese man was admitted to a local hospital with a 14-day history of fever and skin rashes on the elbows and trunk for 1 week, followed by weakness and loss of consciousness. His blood pressure was 124/60 mmHg, and his mental status was assessed on the Glasgow Coma Scale (GCS) as eye-opening score (E) of 2, verbal response score (V) of 2 and motor response score (M) of 3. Laboratory tests showed leukopenia (white blood cell [WBC] count 2.02 × 10^9^/L, lymphocyte count 0.70 × 10^9^/L), increased C-reactive protein (CRP) (4.2 mg/dL), positive results for antinuclear (× 160 speckled) and anti-Smith antibodies (36.2 U/mL), and low levels of serum C3 (53, normal range, 86–160 mg/dL) and C4 (12, normal range, 17–45 mg/dL). No abnormal kidney and liver function findings were observed, and direct Coombs tests were negative. Skin biopsy findings on the trunk were compatible with cutaneous lupus erythematosus. Cerebrospinal fluid (CSF) analysis did not reveal any abnormalities, and no significant abnormal findings on head magnetic resonance imaging (MRI) were observed. The clinical and laboratory findings led to the diagnosis of SLE complicated by neuropsychiatric SLE (NPSLE) based on the European League Against Rheumatism/American College of Rheumatology 2019 classification criteria for SLE (22). The patient was administered methylprednisolone pulse therapy (1 g/day for 3 days), followed by prednisolone 40 mg/day, and his clinical symptoms improved. Six months later, after prednisolone was tapered to 5 mg/day without using any other immunosuppressant, the patient developed the same symptoms that were noted previously, including a history of fever, skin rashes on the elbows and trunk, and loss of consciousness, and was admitted to the same local hospital again. The patient was diagnosed with a relapse of SLE complicated by NPSLE and was started on methylprednisolone pulse therapy (1 g/day for 3 days), followed by prednisolone (60 mg/day); however, his condition did not improve, and he was transferred to our hospital 4 days after admission.

**FIGURE 1:**
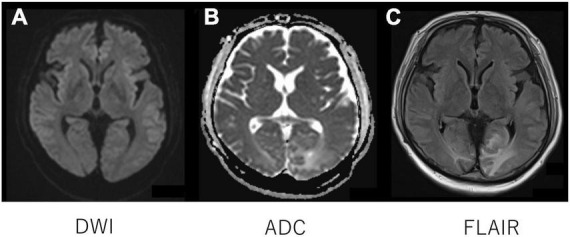
Head magnetic resonance imaging. Diffusion-weighted imaging with MRI demonstrated isointensity **(A)** with an increased signal of apparent diffusion coefficient map **(B)** of the bilateral occipital cortex **(B)**, indicating vasogenic edema in the regions. Fluid-attenuated inversion recovery MRI showed an increased signal intensity in the regions **(C)**.

On admission to our hospital, the patient had a blood pressure of 136/68 mmHg, heart rate of 102 beats/min, oxygen saturation (SpO_2_) of 98% (room air), and body temperature of 39.2°C. The GCS score for his mental status was E2 V1 M2, and maculopapular rashes were observed on the trunk and extremities. Laboratory test results (Table 1) revealed an elevated leukocyte count at 14,000/μL; increased CRP level at 8.59 mg/dL; erythrocyte sedimentation rate of 79 mm/h (normal range, 0–15 mm/h); ferritin level of 11,460/μL; positive results for antinuclear (× 160 speckled) and anti-Smith antibodies (31.9 U/mL); normal levels of serum C3 and C4; elevated aspartate aminotransferase (118 U/L) and hypertriglyceridemia (262 mg/dL); and high level of D-dimer (9.0 mg/dL). Antiphospholipid antibody testing (lupus anticoagulant, and IgG antibodies to cardiolipin and beta2-glycoprotein I) was negative. His kidney functions were normal (urinary protein: 0.12 g/gCr and no hematuria). Bone marrow examination showed infiltration of the bone marrow by activated macrophages, compatible with the findings of MAS (Supplementary Figure 1). Lumbar puncture revealed elevated CSF protein levels at 119 mg/dL (normal range 10–40 mg/dL) and interleukin (IL)-6 levels at 17.3 pg/mL, without elevated WBC count. Tests for tumor cells and bacterial, viral, or fungal infections of the CSF were all negative. Other indicators were negative, including anti-aquaporin-4 antibodies, anti-ribosomal P protein antibodies, and oligoclonal bands. Diffusion-weighted imaging with MRI demonstrated isointensity (Figure 1A) with an increased signal of apparent diffusion coefficient map (Figure 1B) of the bilateral occipital cortex (Figure 1B), indicating vasogenic edema in these regions. Fluid-attenuated inversion recovery MRI showed an increased signal intensity in these regions (Figure 1C).

**TABLE 1:** Main laboratory data.

Variables	Normal values	On admission	Day 15	Day 22 Eculizumab ➀	Day 29 Eculizumab ➁	Day 36
**Hematology tests**						
White blood cells (/μL)	3,300–8,600	140,00	31,00	26,00	46,00	45,00
Lymphocytes (/μL)	1,100–3,200	434	465	260	938	990
Hemoglobin (g/dL)	11.6–14.8	13.8	7.3	7.7	8.2	9.0
Platelets (/μL)	158,000–348,000	172,000	66,000	68,000	240,000	327,000
Schistocytes (/HPF)	Negative	Negative	16	14	14	Negative
Creatinine (mg/dL)	0.46–0.79	0.78	0.58	0.62	0.57	0.73
AST (U/L)	13–30	118	80	153	77	41
ALT (U/L)	10–42	209	70	100	168	74
LDH (U/L)	124–222	538	702	912	407	318
C-reactive protein (mg/dL)	0.0–0.14	8.59	15.11	15.13	2.34	1.85
Ferritin (ng/mL)	31–325	11,460	45,760	56,740	1,843	1,039
PT-INR	0.8–1.2	1.18	1.03	1.07	1.04	1.08
APTT (s) (reference)	24.4–39.2	29.0 (26.7)	29.1 (26.9)	27.5 (26.9)	32.0 (26.9)	33.7 (26.9)
Fibrinogen (mg/dL)	190–330	699	292	162	200	289
D-dimer (μg/dL)	0–1	9.0	11.87	4.53	2.61	1.02
C3 (mg/dL)	86–160	83	73	64	83	89
C4 (mg/dL)	17–45	23.5	17.1	12.2	29.4	31.9
CH50 (U/mL)	30–40	43.8	31	31.9	10.8	11.7
ANA	<40	160 (speckle)				
Anti-dsDNA antibody (U/L)	<12	<10				
Anti-DNA antibody (U/L)	<6.0	<1.7				
Anti-Sm antibody (U/mL)	<10	31.9				
Anti-RNP antibody (U/mL)	<10	32.2				
Anti-SS-A antibody (U/mL)	<10	80.6				
Anti-cardiolipin antibody, IgG	<10	2				
Anti-β2 glycoprotein I	<3.5	<1.3				
Lupus anticoagulant (dRVVT)	1.2	1.00				
ADAMTS13 activity (%)		35	35			
Inhibitor of ADAMTS13			Negative			
**Urinalysis**						
Red blood cells (/HPF)		<4	<4	<4	<4	<4
White blood cells (/HPF)		5–9	<4	<4	<4	<4
Urinary protein (g/gCr)		0.12	0.74	1.10	0.10	0.12

ALT, Alanine transaminase; ANA, Antinuclear antibody; ANCA, Anti-neutrophil cytoplasmic antibody; APTT, Activated partial thromboplastin time; AST, Aspartate transaminase; dRVVT, diluted Russell viper venom time; HPF, High-power field; LDH, Lactate dehydrogenase; MPO, Myeloperoxidase; PT, Prothrombin time; PR3, Proteinase3; HPF, high-power field.

**FIGURE 2:**
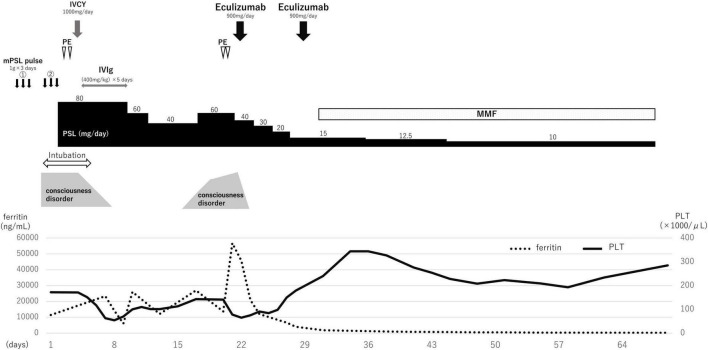
Clinical course.

Based on the clinical features, SLE complicated by NPSLE, and SLE-associated MAS was diagnosed. With tachypnea at 40 breaths/min, the patient required mechanical ventilation. A second methylprednisolone pulse therapy (1 g/day for 3 days) was administered, with subsequent administration of 80 mg/day prednisolone, 1,000 mg intravenous cyclophosphamide (IVCY) once, and 0.4 g/kg/day intravenous immunoglobulin (IVIg) for 5 days (Figure 2). Two concomitant plasma exchanges (one per day) were performed with fresh-frozen plasma. Once the general condition and state of consciousness had improved gradually (but had not fully recovered), the patient was de-intubated 5 days after admission. However, his state of consciousness worsened with a high fever, and blood tests revealed elevated inflammation findings, such as higher levels of CRP (15.11 mg/dL) and ferritin (45,760/μL). Furthermore, hemolytic anemia (hemoglobin 7.3 g/dL) with a new appearance of peripheral schistocytes, and high lactate dehydrogenase levels (702 U/L) were found on day 15 after admission. Regarding kidney function, the patient had a normal creatinine level (0.58 mg/dL) but mild proteinuria (urinary protein: 0.74 g/gCr). The infection survey, including blood culture, cytomegalovirus-antigenemia, and computed tomography scan was negative, and no remarkable change was observed on the head MRI. According to the clinical diagnosis, TMA concomitant with MAS was suspected. *Escherichia coli* O157:H7 infection was excluded on stool examination. ADAMTS13 activity was normal (activity: 35%), eliminating thrombotic thrombocytopenic purpura (TTP). Subsequently, we performed a plasma exchange again on day 20 after admission; however, the patient’s condition worsened. On day 22 after admission, eculizumab treatment was initiated. One day after eculizumab administration, the patient’s mental status improved significantly, followed by an excellent hematological response. Urinary protein also decreased from 1.10 g/gCr at the first administration of eculizumab to 0.10 g/gCr after 7 days following the first administration of eculizumab, suggesting that the patient might have had mild renal involvement associated with TMA. In total, two doses of eculizumab were administered every week for 2 weeks, and steroid doses were decreased rapidly. Mycophenolate mofetil (2,000 mg/day) and hydroxychloroquine (300 mg/day) were administered 30 days after admission and the prednisolone dosage was tapered to 5 mg/day on day 102 after admission.

In the present case, we evaluated the changes in serum syndecan-1 and hyaluronan, which are components of vascular endothelial cells, and soluble C5b-9 (sC5b-9), which is a terminal product of the complement system and indicative of complement activation, before and after eculizumab administration (Table 2), as biomarkers for activating the complement system. In addition, we evaluated those of kidney donor candidates (*n* = 5) considered as healthy controls (Supplementary Table 1). Serum syndecan-1 and hyaluronan were measured using the Duoset ELISA kit [(Diaclone, France), and (PG research, Japan), respectively] and sC5b-9 levels were measured using the MicroVue SC5b-9 Plus ELISA kit (Quidel Corp., America).

**TABLE 2:** Changes in syndecan-1, hyaluronan, and sC5b-9 levels before and after eculizumab administration.

	Healthy control (range)	On admission	Day 22 Eculizumab ➀	Day 29 Eculizumab ➁	Day 36	Day 43	Day 50
Syndecan-1 (ng/mL)	7.5–29.0	551.976	934.931	563.81	297.835	119.774	69.005
Hyaluronan (ng/mL)	5.5–26.2	180.78	115.936	38.21	20.143	24.541	20.81
sC5b-9 (ng/mL)	458–1,171	3476.137	1804.498	4641.998	2601.567	1117.298	3338.627

sC5b-9, soluble C5b-9.

As a result, serum syndecan-1, hyaluronan, and sC5b-9 levels before eculizumab administration were elevated above those of healthy controls (Table 2). However, after eculizumab administration, serum syndecan-1 and hyaluronan levels decreased rapidly to normal, with concomitant improvement in clinical symptoms (Table 2), suggesting that TMA was the primary pathogenesis of the patient. Meanwhile, sC5b-9 levels showed no remarkable change before and after eculizumab administration.

Genetic mutation analysis did not identify any known pathogenic mutation in complement regulatory genes associated with complement-mediated aHUS, which is now recognized. A follow-up MRI, one year after admission, showed persistent abnormal findings in the brain, indicating that damage due to vasogenic edema could ultimately result in permanent injury to the brain parenchyma. However, no abnormal neurological symptoms, subsequent relapse, or adverse events occurred during the follow-up.

## 3. Discussion

To the best of our knowledge, this is the first reported case of TMA concomitant with SLE-associated MAS that rapidly recovered after only two doses of eculizumab and was refractory to conventional treatment with high-dose steroids, IVCY, and plasma exchange. In the present case, we confirmed that serum syndecan-1 and hyaluronan levels, biomarkers of endothelial damage, decreased after eculizumab administration. This case illustrates that it is essential to appropriately assess the possibility of TMA in diagnosing SLE-associated MAS and consider the use of eculizumab as a therapeutic option.

The pathogenesis of TMA in SLE remains unclear and may be multifactorial (23, 24). Since SLE is an immune complex-mediated disease, it is suggested that activation of the classical pathway plays a key role in the development of TMA. However, some studies have shown that dysregulation of the alternative complement pathway may also be involved (25). There is a hypothesis that the activation of the alternative pathway may play a role in complement activation-induced self-injury and inflammatory response, leading to TMA in SLE (26). Several previous studies have reported that eculizumab is an effective treatment for complement-mediated TMA (18–20). Other reports have also revealed that in cases of lupus nephritis complicated by TMA refractory to conventional immunosuppressive treatment, eculizumab seems to be an efficacious therapy (21, 23, 25–27). It is believed that once complement overactivation occurs, especially activation of the alternative complement pathway, in endothelial cells and TMA develops, conventional immunosuppressive treatment may neither suppress the complement pathway nor stop TMA progression (23). Even in a healthy host, severe glomerular endothelial injuries may lead to exhausted complement activation, damage to the membrane complement regulators, and further tissue injuries, as described in our previous report (28). Therefore, although no previous reports have assessed the efficacy of eculizumab for refractory TMA concomitant with SLE-associated MAS, we reasoned that it might be a feasible strategy to use eculizumab treatment in such patients.

However, it is challenging to recognize TMA in a timely manner in the diagnosis of MAS because the clinical characteristics of TMA are similar to those of MAS (8). To identify TMA in the diagnosis of MAS, an accurate and sensitive assessment of endothelial damage should be made for each patient with SLE. However, this approach is not yet fully established. Hence, in the present case, we focused on serum syndecan-1 and hyaluronan, and sC5b-9, which is a terminal product of the complement system and indicative of complement activation, as biomarkers for detecting TMA and evaluated the changes in them before and after eculizumab administration (Table 2).

Levels of serum syndecan-1 and hyaluronan correlate or become elevated in various diseases, including sepsis, trauma, TTP, and SLE with and without lupus nephritis (29–33). We recently reported that serum levels of hyaluronan were useful in assessing vascular endothelial injuries, such as pre-eclampsia (34). This indicates that serum syndecan-1 and hyaluronan levels reflect the degree of endothelial cell damage. In the patient described in the present study, serum syndecan-1 and hyaluronan levels decreased rapidly to normal after eculizumab administration, with concomitant improvement in clinical symptoms. This result suggests that the patient’s condition was mainly due to vascular endothelial cell damage, namely TMA, and that eculizumab could contribute to suppressing the progression of endothelial cell damage by controlling the over-activation of the complement system.

sC5b-9 is also reported to be increased in various diseases where tissue damage subsequently activates the complement system. These include complement-mediated TMA, TTP, sepsis, lupus nephritis, and others (35, 36). However, other reports show that sC5b-9 does not always correlate with TMA disease activity because it does not necessarily represent the amount of C5b-9 formed on the tissue (37, 38). In our case, although the patient’s clinical symptoms improved rapidly after eculizumab administration, sC5b-9 remained at a high level for up to 50 days and there was a discrepancy between clinical symptoms and the sC5b-9 level (Table 2). Therefore, although sC5b-9 may not always correlate with TMA status in SLE patients, it is difficult to show any evidence at this moment. Further studies should be undertaken to clarify the mechanisms involved.

Regarding optimization of eculizumab usage for treating TMA in SLE, although the optimal dose and period of administration has not been determined by us, eculizumab was used only twice during the extreme phase of TMA in our case, after which the steroid dose could be reduced early, and remission was sustained by using only conventional immunosuppressive treatment. The patient might have been trapped in a vicious cycle of complement overactivation after developing TMA and when conventional immunosuppressive therapy failed to stop complement activation. Specifically, it was shown that eculizumab was significantly effective since the main cause of complement overactivation was considered endothelial damage. In addition, no genetic abnormality of the complement system was observed, suggesting that the patient achieved remission after only two doses of eculizumab, and remission was sustained using only conventional immunosuppressive treatment. Furthermore, serum syndecan-1 and hyaluronan levels decreased and remained at low levels after eculizumab administration. If serum syndecan-1 levels have decreased to normal levels and remain at decreased levels after eculizumab administration, then this may be a clinically appropriate time to decide whether eculizumab can be discontinued or not. Therefore, the utility of biomarkers, including serum syndecan-1 and hyaluronan levels, for managing TMA-related diseases should now be assessed to specify the optimal strategy of eculizumab for SLE-associated TMA.

As to whether our case was complement-mediated TMA or SLE-related TMA, it was difficult to differentiate between the two disorders because the symptoms of complement-mediated TMA and autoimmune disorders sometimes overlap (39), and the presence of underlying genetic variants in complement genes is rare in patients with SLE (40). Our patient had no disease-causing mutations in the genes of the complement components; however, their regulators have not been evaluated in approximately 50% of clinically suspected complement-mediated TMA cases (41, 42). Therefore, the presence of unscreened complement-related pathogenic genetic mutations cannot be ruled out. However, in complement-mediated TMA, patients may be treated for an extended period to suppress TMA (40), while autoimmune disease-related TMA appears as a non-relapsing form of TMA (38). In our case, the TMA improved after only two administrations of eculizumab, and subsequent remission was sustained. Therefore, considering our course of clinical treatment, we believe that overt complement activation was induced by SLE rather than by complement-mediated TMA.

Finally, the pathological cause of the co-occurring TMA and MAS was unclear in our case. It is possible that high levels of inflammatory cytokines (type I and II interferon) in MAS may have injured the endothelium directly, subsequently activating the complement system, and leading to the development of TMA (43–47). Further studies should be undertaken to clarify such possibilities.

## 4. Conclusion

We report the first case of TMA concomitant with SLE-associated MAS that was refractory to conventional immunosuppressive therapy but showed a remarkable recovery after eculizumab treatment. The present case suggests that conventional immunosuppressive treatment may not suppress vascular endothelial damage when TMA is complicated by complement activation in SLE. In such a situation, eculizumab may promptly stop the complement overactivation in vascular endothelial cells. Furthermore, there is a possibility of utilizing biomarkers of vascular endothelial damage, such as syndecan-1 and hyaluronan, to assess the efficacy of eculizumab treatment and set the treatment duration, and this prospect requires further validation.

## Data availability statement

The original contributions presented in this study are included in this article/Supplementary material, further inquiries can be directed to the corresponding author.

## Ethics statement

The studies involving human participants were reviewed and approved by the Ethics Committees for Human Research at Aichi Medical University Hospital (approval number: 2020-017). The patients/participants provided their written informed consent to participate in this study. Written informed consent was obtained from the individual(s) for the publication of any potentially identifiable images or data included in this article.

## Author contributions

MY and YI contributed to the study design. TI, TK, AA, SI, HK, HN, FK, SB, MM, and YI contributed to the implementation and supervision of the study. All authors participated in the writing of the report and took full responsibility for all aspects of the study and the final manuscript.

## Supplementary material

Supplementary Figure 1 Bone marrow evaluation. Reactive histocytes show phagocytosis of red blood cells (red arrow). May–Giemsa stain (×400).

## References

[1] Veyradier A Meyer D. Thrombotic thrombocytopenic purpura and its diagnosis. *J Thromb Haemost.* (2005) 3:2420–7. 10.1111/j.1538-7836.2005.01350.x 15892859

[2] Jain R Chartash E Susin M Furie R. Systemic lupus erythematosus complicated by thrombotic microangiopathy. *Semin Arthritis Rheum.* (1994) 24:173–82. 10.1016/0049-0172(94)90073-6 7899875

[3] Kello N Khoury LE Marder G Furie R Zapantis E Horowitz DL. Secondary thrombotic microangiopathy in systemic lupus erythematosus and antiphospholipid syndrome, the role of complement and use of eculizumab: case series and review of literature. *Semin Arthritis Rheum.* (2019) 49:74–83. 10.1016/j.semarthrit.2018.11.005 30598332

[4] Noda S Ogura M Tsutsumi A Udagawa T Kamei K Matsuoka K Thrombotic microangiopathy due to multiple autoantibodies related to antiphospholipid syndrome. *Pediatr Nephrol.* (2012) 27:681–5. 10.1007/s00467-011-2085-5 22210384

[5] Muscal E Edwards RM Kearney DL Hicks JM Myones BL Teruya J. Thrombotic microangiopathic hemolytic anemia with reduction of ADAMTS13 activity: initial manifestation of childhood-onset systemic lupus erythematosus. *Am J Clin Pathol.* (2011) 135:406–16. 10.1309/AJCP5BVL4FCLCGLU 21350095

[6] Brunner HI Freedman M Silverman ED. Close relationship between systemic lupus erythematosus and thrombotic thrombocytopenic purpura in childhood. *Arthritis Rheum.* (1999) 42:2346–55. 10555030 10.1002/1529-0131(199911)42:11<2346::AID-ANR13>3.0.CO;2-X

[7] Babar F Cohen SD. Thrombotic microangiopathies with rheumatologic involvement. *Rheum Dis Clin North Am.* (2018) 44:635–49. 10.1016/j.rdc.2018.06.010 30274628

[8] Ravelli A Davı S Minoia F Martini A Cron RQ. Macrophage activation syndrome. *Hematol Oncol Clin North Am.* (2015) 29:927–41. 10.1016/j.hoc.2015.06.010 26461152

[9] Ravelli A Grom AA Behrens EM Cron RQ. Macrophage activation syndrome as part of systemic juvenile idiopathic arthritis: diagnosis, genetics, pathophysiology and treatment. *Genes Immun.* (2012) 13:289–98. 10.1038/gene.2012.3 22418018

[10] Grom AA Horne A De Benedetti F. Macrophage activation syndrome in the era of biologic therapy. *Nat Rev Rheumatol.* (2016) 12:259–68. 10.1038/nrrheum.2015.179 27009539 PMC5851441

[11] Vilaiyuk S Sirachainan N Wanitkun S Pirojsakul K Vaewpanich J. Recurrent macrophage activation syndrome as the primary manifestation in systemic lupus erythematosus and the benefit of serial ferritin measurements: a case-based review. *Clin Rheumatol.* (2013) 32:899–904. 10.1007/s10067-013-2227-1 23483294

[12] Borgia RE Gerstein M Levy DM Silverman ED Hiraki LT. Features, treatment, and outcomes of macrophage activation syndrome in childhood-onset systemic lupus erythematosus. *Arthritis Rheumatol.* (2018) 70:616–24. 10.1002/art.40417 29342508

[13] Lerkvaleekul B Vilaiyuk S. Macrophage activation syndrome: early diagnosis is key. *Open Access Rheumatol.* (2018) 10:117–28. 10.2147/OARRR.S151013 30214327 PMC6124446

[14] Crayne CB Albeituni S Nichols KE Cron RQ. The immunology of macrophage activation syndrome. *Front Immunol.* (2019) 10:119. 10.3389/fimmu.2019.00119 30774631 PMC6367262

[15] Jodele S Dandoy CE Myers KC El-Bietar J Nelson A Wallace G New approaches in the diagnosis, pathophysiology, and treatment of pediatric hematopoietic stem cell transplantation-associated thrombotic microangiopathy. *Transfus Apher Sci.* (2016) 54:181–90. 10.1016/j.transci.2016.04.007 27156964 PMC5710737

[16] Jodele S Laskin BL Dandoy CE Myers KC El-Bietar J Davies SM A new paradigm: diagnosis and management of HSCT- associated thrombotic microangiopathy as multi-system endothelial injury. *Blood Rev.* (2015) 29:191–204. 10.1016/j.blre.2014.11.001 25483393 PMC4659438

[17] Chiurchiu C Ruggenenti P Remuzzi G. Thrombotic microangiopathy in renal transplantation. *Ann Transplant.* (2002) 7:28–33. 12221901

[18] Minoia F Tibaldi J Muratore V Gallizzi R Bracaglia C Arduini A Thrombotic microangiopathy associated with macrophage activation syndrome: a multinational study of 23 patients. *J Pediatr.* (2021) 235:196–202. 10.1016/j.jpeds.2021.04.004 33836183

[19] Gloude NJ Dandoy CE Davies SM Myers KC Jordan MB Marsh RA Thinking beyond HLH: clinical features of patients with concurrent presentation of hemophagocytic lymphohistiocytosis and thrombotic microangiopathy. *J Clin Immunol.* (2020) 40:699–707. 10.1007/s10875-020-00789-4 32447592 PMC7245179

[20] Moake JL. Thrombotic microangiopathies. *N Engl J Med.* (2002) 347:589–600. 10.1056/NEJMra020528 12192020

[21] George JN Nester CM. Syndromes of thrombotic microangiopathy. *N Engl J Med.* (2014) 371:654–66. 10.1056/NEJMra1312353 25119611

[22] Legendre CM Licht C Muus P Greenbaum LA Babu S Bedrosian C Terminal complement inhibitor eculizumab in atypical hemolytic–uremic syndrome. *N Engl J Med.* (2013) 368:2169–81. 10.1056/NEJMoa1208981 23738544

[23] Wright RD Bannerman F Beresford MW Oni L. A systematic review of the role of eculizumab in systemic lupus erythematosus-associated thrombotic microangiopathy. *BMC Nephrol.* (2020) 21:245. 10.1186/s12882-020-01888-5 32605540 PMC7329551

[24] Aringer M Costenbader K Daikh D Brinks R Mosca M Ramsey-Goldman R 2019 European league against rheumatism/American college of rheumatology classification criteria for systemic lupus erythematosus. *Ann Rheum Dis.* (2019) 78:1151–9. 10.1136/annrheumdis-2018-214819 31383717

[25] Song D Wu LH Wang FM Yang XW Zhu D Chen M The spectrum of renal thrombotic microangiopathy in lupus nephritis. *Arthritis Res Ther.* (2013) 15:R12. 10.1186/ar4142 23320601 PMC3672792

[26] Moschetti L Piantoni S Vizzardi E Sciatti E Riccardi M Franceschini F Endothelial dysfunction in systemic lupus erythematosus and systemic sclerosis: a common trigger for different microvascular diseases. *Front Med.* (2022) 9:849086. 10.3389/fmed.2022.849086 35462989 PMC9023861

[27] Azevedo A Faria B Teixeira C Carvalho F Neto G Santos J Portuguese consensus document statement in diagnostic and management of atypical hemolytic uremic syndrome. *Port J Nephrol Hypert.* (2018) 32:1–22.

[28] de Holanda MI Pôrto LC Wagner T Christiani LF Palma LMP. Use of eculizumab in a systemic lupus erythemathosus patient presenting thrombotic microangiopathy and heterozygous deletion in CFHR1-CFHR3. A case report and systematic review. *Clin Rheumatol.* (2017) 36:2859–67. 10.1007/s10067-017-3823-2 28905254

[29] Pickering MC Ismajli M Condon MB McKenna N Hall AE Lightstone L Eculizumab as rescue therapy in severe resistant lupus nephritis. *Rheumatology.* (2015) 54:2286–8. 10.1093/rheumatology/kev307 26316577 PMC4643725

[30] Mizuno M Nozaki M Morine N Suzuki N Nishikawa K Morgan BP A protein toxin from the sea anemone *Phyllodiscus semoni* targets the kidney and causes a renal injury resembling haemolytic uremic syndrome. *Am J Pathol.* (2007) 171:402–14. 17600120 10.2353/ajpath.2007.060984 PMC1934535

[31] Paulus P Jennewein C Zacharowski K. Biomarkers of endothelial dysfunction: can they help us deciphering systemic inflammation and sepsis? *Biomarkers.* (2011) 16:S11–21. 10.3109/1354750X.2011.587893 21707440

[32] Woodcock TE Woodcock TM. Revised starling equation and the glycocalyx model of transvascular fluid exchange: an improved paradigm for prescribing intravenous fluid therapy. *Br J Anaesth.* (2012) 108:384–94. 10.1093/bja/aer515 22290457

[33] Frati-Munari AC. Medical significance of endothelial glycocalyx. *Arch Cardiol Mex.* (2013) 83:303–12. 10.1016/j.acmx.2013.04.015 24280179

[34] Lee WL Slutsky AS. Sepsis and endothelial permeability. *N Engl J Med.* (2010) 363:689–91. 10.1056/NEJMcibr1007320 20818861

[35] Kim KJ Kim JY Baek IW Kim WU Cho CS. Elevated serum levels of syndecan-1 are associated with renal involvement in patients with systemic lupus erythematosus. *J Rheumatol.* (2015) 42:202–9. 10.3899/jrheum.140568 25512478

[36] Asai A Hatayama N Kamiya K Yamauchi M Kinashi H Katsuno T Roles of glomerular endothelial hyaluronan in the development of proteinuria. *Physiol Rep.* (2021) 9:e15019. 10.14814/phy2.15019 34472715 PMC8411502

[37] Sperati CJ. How I treat complement-mediated TMA. *Clin J Am Soc Nephrol.* (2022) 17:452–4. 10.2215/CJN.13581021 35074846 PMC8975032

[38] Song D Guo WY Wang FM Li YZ Song Y Yu F Complement alternative pathways activation in patients with lupus nephritis. *Am J Med Sci.* (2017) 353:247–57. 28262211 10.1016/j.amjms.2017.01.005

[39] Noris M Galbusera M Gastoldi S Macor P Banterla F Bresin E Dynamics of complement activation in aHUS and how to monitor eculizumab therapy. *Blood.* (2014) 124:1715–26. 10.1182/blood-2014-02-558296 25037630 PMC4162105

[40] Koopman JJE van Essen MF Rennke HG de Vries APJ van Kooten C. Deposition of the membrane attack complex in healthy and diseased human kidneys. *Front Immunol.* (2021) 11:599974. 10.3389/fimmu.2020.599974 33643288 PMC7906018

[41] Dellal A Bige N Hilliquin P Boffa JJ Rondeau E Hatron PY Thrombotic microangiopathy associated with anti-neutrophil cytoplasmic antibody-associated vasculitis: a French nationwide retrospective case-control study and literature review. *Rheumatology.* (2019) 58:1873–5. 10.1093/rheumatology/kez167 31330026

[42] Le Clech A Simon-Tillaux N Provôt F Delmas Y Vieira-Martins P Limou S Atypical and secondary hemolytic uremic syndromes have a distinct presentation and no common genetic risk factors. *Kidney Int.* (2019) 95:1443–52. 10.1016/j.kint.2019.01.023 30982675

[43] Loirat C Noris M Fremeaux-Bacchi V. Complement and the atypical hemolytic uremic syndrome in children. *Pediatr Nephrol.* (2008) 23:1957–72. 10.1007/s00467-008-0872-4 18594873 PMC6904381

[44] Brodsky RA. Eculizumab and aHUS: to stop or not. *Blood.* (2021) 137:2419–20. 10.1182/blood.2020010234 33956069 PMC8109016

[45] Kavanagh D McGlasson S Jury A Williams J Scolding N Bellamy C Type I interferon causes thrombotic microangiopathy by a dose-dependent toxic effect on the microvasculature. *Blood.* (2016) 128:2824–33. 10.1182/blood-2016-05-715987 27663672 PMC5159705

[46] Hunt D Kavanagh D Drummond I Weller B Bellamy C Overell J Thrombotic microangiopathy associated with interferon beta. *N Engl J Med.* (2014) 370:1270–1. 10.1056/NEJMc1316118 24670186 PMC4066182

[47] Bracaglia C De Graaf K Marafon DP Guilhot F Ferlin W Prencipe G Elevated circulating levels of interferon-γ and interferon-γ-induced chemokines characterize patients with macrophage activation syndrome complicating systemic juvenile idiopathic arthritis. *Ann Rheum Dis.* (2017) 76:166–72. 10.1136/annrheumdis-2015-209020 27296321

