# Supplementary material for: Case report: Thrombotic microangiopathy concomitant with macrophage activation syndrome in systemic lupus erythematosus refractory to conventional treatment successfully treated with eculizumab
Source: Front Med (Lausanne). 2023 Jan 9;9:1097528. doi: 10.3389/fmed.2022.1097528 (PMC9868404; doi:10.3389/fmed.2022.1097528)
Supplement: Supplementary file 1 [file Table_1.docx]

**Supplementary table 1. Main data of healthy control (n = 5)**

|  | **N = 5** |
| --- | --- |
| Age (year) | 52 (38–77) |
| Male sex | 3 (60.0) |
| eGFR (mL/min/1.73 m^2^) | 78 (55–100) |
| Syndecan-1 (ng/mL) | 11.2 (7.5–29.0) |
| Hyaluronan (ng/mL) | 13.2 (5.5–26.2) |
| sC5b-9 (ng/mL) | 614 (458–1171) |

Continuous data are presented as medians (ranges), while categorical data isre expressed as numbers (proportions).
