# Supplementary figures and images for: Case report: Thrombotic microangiopathy concomitant with macrophage activation syndrome in systemic lupus erythematosus refractory to conventional treatment successfully treated with eculizumab
Source: Front Med (Lausanne). 2023 Jan 9;9:1097528. doi: 10.3389/fmed.2022.1097528 (PMC9868404; doi:10.3389/fmed.2022.1097528)

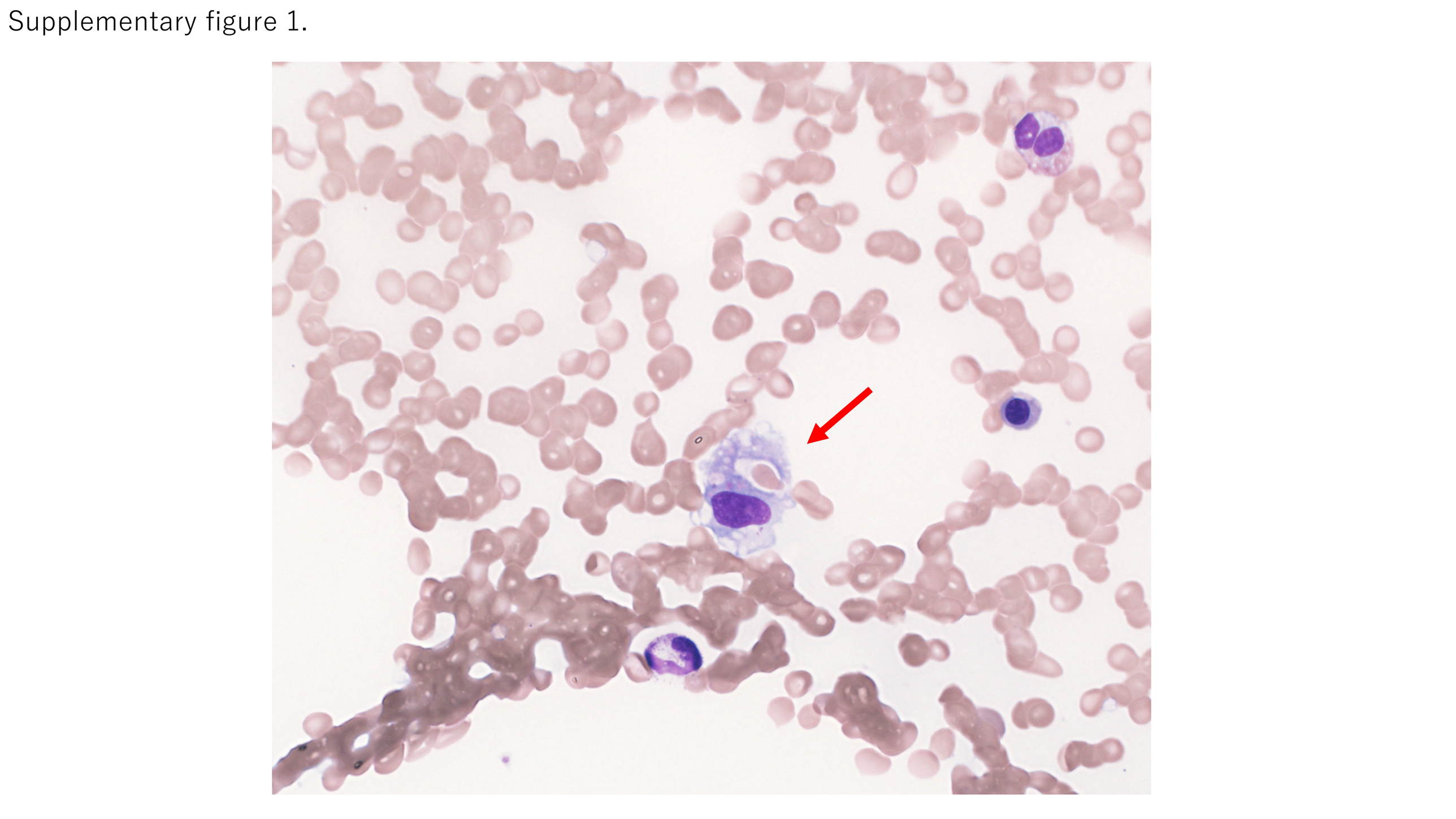

Supplement: Supplementary Figure 1 — Bone marrow evaluation. Reactive histocytes show phagocytosis of red blood cells (red arrow). May–Giemsa stain (×400). [file Image_1.JPEG]
